# An eco-friendly and cost-effective HPTLC method for quantification of COVID-19 antiviral drug and co-administered medications in spiked human plasma

**DOI:** 10.1038/s41598-024-56923-4

**Published:** 2024-05-01

**Authors:** Ekram A. Ghozzy, Nahed M. El-Enany, Manar M. Tolba, Samah Abo El Abass

**Affiliations:** 1 Pharmaceutical Chemistry Department, Faculty of Pharmacy, Delta University for Science and Technology, Gamasa, 35712 Egypt; 2 Pharmaceutical Analytical Chemistry Department, Faculty of Pharmacy, Mansoura University, Mansoura, 35516 Egypt; 3 Pharmaceutical Chemistry Department, Faculty of Pharmacy, New Mansoura University, New Mansoura, 7723730 Egypt

**Keywords:** COVID-19 antiviral, Remdesivir, TLC, Spiked human plasma, The method’s greenness, Drug safety, Drug screening

## Abstract

The coronavirus-2 has led to a global pandemic of COVID-19 with an outbreak of severe acute respiratory syndrome leading to worldwide quarantine measures and a rise in death rates. The objective of this study is to propose a green, sensitive, and selective densitometric method to simultaneously quantify remdesivir (REM) in the presence of the co-administered drug linezolid (LNZ) and rivaroxaban (RIV) in spiked human plasma. TLC silica gel aluminum plates 60 F254 were used as the stationary phase, and the mobile phase was composed of dichloromethane (DCM): acetone (8.5:1.5, v/v) with densitometric detection at 254 nm. Well-resolved peaks have been observed with retardation factors (R_f_) of 0.23, 0.53, and 0.72 for REM, LNZ, and RIV, respectively. A validation study was conducted according to ICH Q2 (R1) Guidelines. The method was rectilinear over the concentration ranges of 0.2–5.5 μg/band, 0.2–4.5 μg/band and 0.1–3.0 μg/band for REM, LNZ and RIV, respectively. The sensitivities of REM, LIN, and RIV were outstanding, with quantitation limits of 128.8, 50.5, and 55.8 ng/band, respectively. The approach has shown outstanding recoveries ranging from 98.3 to 101.2% when applied to pharmaceutical formulations and spiked human plasma. The method’s greenness was assessed using Analytical Eco-scale, GAPI, and AGREE metrics.

## Introduction

By the end of 2019, abnormal pneumonia outbreak associated with a new coronavirus had begun in Wuhan City, China. Afterwards it was termed Covid-19 by The World Health Organization (WHO). The disease spread so rapidly that the WHO declared it a pandemic in March 2020^1^. The main feature of SARS-CoV-2 is its nonspecific symptoms, which are frequently misdiagnosed with flu and common cold^2^.

The other troubling aspect of this condition is its unknown prognosis, which can result in deadly outcomes like pneumonia and acute respiratory syndrome, or produce none to mild respiratory tract complaints which will subside just with supportive care. Patients with diabetes, chronic respiratory and cardiovascular disorders, cancer, and immune system deficiency may encounter serious pathological complications that result in fatality^3^.

In the absence of a specific antiviral medication against SARS-CoV-2, along with the failure of mass vaccination to halt the pandemic^4^, studies were carried out to evaluate the efficiency of existing antiviral agents targeting SARS-Cov2 such as remdesivir^5^.

**Figure 1:**
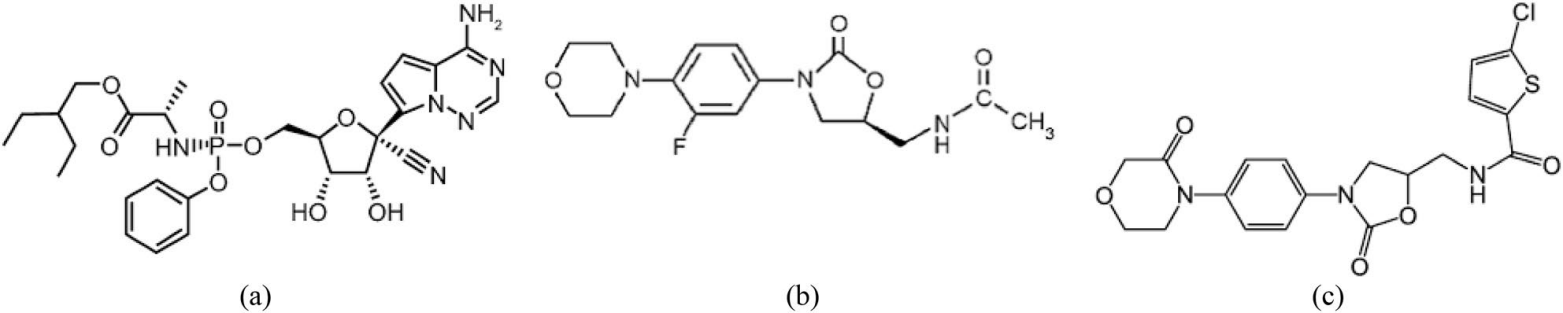
Chemical Structures of the investigated drugs; (**a**) remdesivir, (**b**) linezolid, (**c**) rivaroxaban.

Remdesivir (REM) is 2-ethylbutyl (2S)-2-[[[(2R,3S,4R,5R)-5-(4-amino pyrrolo[2,1-f][1,2,4]triazin-7-yl)-5cyano3,4dihydroxyoxolanyl]methoxyphenoxy phosphoryl] amino] propanoate, (Fig. 1a). It was originally developed as an Ebola virus treatment by Gilead Sciences^6^.

Remdesivir is a nucleotide analog prodrug, that disrupts the viral RNA synthesis by being bioactivated into GS-441524 and phosphorylated into an active nucleoside triphosphate metabolite^7^.

Studies explored REM’s effects on SARS-CoV-2 as a potential COVID-19 treatment. The FDA approved REM as the sole antiviral treatment for COVID-19 due to its ability to block the virus’s replication^8^.

The FDA has accorded REM an “emergency use authorization” for severe COVID-19 since May 1, 2020, though few analytical methods has been reported for the estimation of remdesivir including, liquid chromatographic techniques^9–17^, electrochemical approaches^18^, spectrophotometric^17,19^ and spectrofluorimetric methods^20–22^. Most already elaborated methods are time-consuming, require expensive analytical tools, hazardous chemicals, and skilled personnel. The present work aimed to determine REM using a simple analytical procedure.

In order to limit the viral replication and improve the patients’ health, COVID-19 treatment involved the administration of many therapeutics from various categories, trying many preexisting medicines^23^. Therefore, a quick, valid, and reasonably priced bioanalytical approach for REM measurement in the human plasma matrix is urgently required for use in further clinical investigation and therapeutic drug monitoring.

The treatment guidance includes the use of broad-spectrum antibiotics for the treatment of secondary bacterial infection and acquired pneumonia^24^. Linezolid (LNZ) is (S)-N-[[3-[3-Fluoro-4-(4-morpholinyl)phenyl]-2-oxo-5-oxazolidinyl] methyl] acetamide (Fig. 1b), an antibiotic, was effective in treating COVID-19 patients with bacterial pneumonia, linezolid’s superiority is a result of its better penetration into the respiratory secretion compared to other broad-spectrum antibiotics, particularly vancomycin^25^. In addition, molecular docking studies have demonstrated the interaction between linezolid and the SARS-CoV-2 spike protein, indicating the stability and potential activity of linezolid on the SARS-CoV-2 protein^26^.

Anticoagulants are also included in the treatment protocol to reduce the risk of thrombosis, which is observed in nearly all COVID-19 patients^27^. Rivaroxaban (RIV) is (S)-5-chloro-N-{[2-oxo-3-[4-(3-oxomorpholin-4-yl) phenyl]oxazolidin-5-yl]methyl}vthiophene-2-carboxamide (Fig. 1c), which reduces the risk of venous thromboembolism without significantly raising the risk of severe bleeding, resulting in a net therapeutic benefit. This benefit seems to be more noticeable in patients whose initial hospitalization was due to an infectious condition, particularly pneumonia^28^.

The US Food and Drug Administration (FDA) granted authorization to remdesivir, as the initial antiviral medication approved for treating COVID-19. Antibiotics, antipyretics, corticosteroids, and anticoagulants are also included in the treatment protocol, which primarily relies on remdesivir. There is currently no reported method for simultaneously analyzing REM, LNZ, and RIV in biological fluids as co-administered medications. Thus, the current study aims at developing a simple, cost-effective, and eco-friendly analytical technique for estimating the licensed COVID-19 antiviral drug REM in plasma in the presence of routinely used drugs in corona virus treatment: antibiotics such as LNZ and anticoagulants such as RIV.

## Experimental

### Apparatus

- CAMAG^®^ TLC scanner 3 (CAMAG, Muttenz, Switzerland) and winCATS^®^ software. The scanner uses a deuterium lamp as the radiation source with scanning speed of 20 mm/s. Absorbance mode was utilized as the scan mode and the output is a chromatogram and an integrated peak area.
- Linomat 5 autosampler with 100 µL microsyringe (CAMAG, Muttenz, Switzerland)
- TLC Silica gel 60 F254 (Aluminum sheets 20 × 20 cm, 0.1 mm thickness, Merck, Darmstadt, Germany).
- Vortex mixer, ZX3 (Velp. Scientifca, Usmate, Italy).
- Centrifuge, model 2-16P (Sigma, Osterode am Harz, Germany).

### Materials and reagents

- Remdesivir reference substance (Purity 99.8% as certified) was generously gifted by EIPICo. 10th of Ramadan City, Egypt.
- Linezolid reference substance (Purity 99.8% as certified) was kindly provided by Averroes Pharma, 6th Industrial Zone Sadat City, Egypt.
- Rivaroxaban reference substance (Purity 99.6% as certified) was kindly supplied as a gift sample from AChemBlock, Burlingame, USA.
- HPLC grade methanol, ethanol and ethyl acetate were purchased from Fisher Scientifics, Belgium.
- Dichloromethane of analytical grade was obtained from Pioneers for chemicals (Piochem, 6th October City, Egypt).
- Human plasma samples were supplied by Mansoura University Hospital, Mansoura, Egypt, and kept in the freezer at − 20 °C until usage.

### Pharmaceutical dosage forms

Remdesivir-Rameda^®^ concentrate for solution for I.V infusion, batch number 214188 (Rameda Pharmaceuticals, 6th October City, Giza, Egypt) stated to contain 100.0 mg REM/vial, was obtained from the Egyptian market.

Linezolid^®^ solution for IV infusion, batch number 21080023 (Global NAPI Pharmaceuticals, 6th October City, Giza, Egypt), was obtained from the Egyptian market and was noted to contain 200.0 mg/100 mL-vial.

Xarelto^®^ film coated tablets (Bayer Schering Pharma, batch no. 04008500074763) were bought as well from the Egyptian market and stated to contain 10.0 mg/tablet of rivaroxaban.

### Standard solutions

Accurately weighed amounts equivalent to 10.0 mg of REM, LNZ and RIV were put into three separate 10 mL volumetric flask then the volumes were brought up to the required volume by methanol for REM and LNZ and acetonitrile for RIV to produce 1.0 mg/mL standard solutions. The produced stock solutions remained stable for 14 days in the refrigerator without changing.

### Procedures

#### Chromatographic conditions

Following the saturation of the chromatographic jar for 30 min by the eluent system; dichloromethane–acetone (8.5:1.5, v/v). At 1.0 cm from the bottom edge, the sample bands were applied. Subsequently, chromatographic development and air drying was performed. This was followed by scanning the plates at 254 nm.

#### Calibration graphs construction

In methanol, REM, LNZ, and RIV concentration sets were prepared, with concentrations ranging from 20 to 550, 20 to 450, and 10 to 300 µg/mL, respectively. In triplicates, 10 µL aliquots of each flask were spotted on TLC plates to obtain concentration ranges of 0.2–5.5, 0.2–4.5, and 0.1–3.0 µg/band of REM, LNZ, and RIV, respectively. By graphing concentrations (µg/band) *versus* the relevant areas under the peaks, calibration plots were constructed.

#### Pharmaceutical dosage forms analysis

An accurately measured volume of the Remdesivir-Rameda^®^ concentrate for solution for I.V infusion equivalent to 10.0 mg was transferred into a 10-mL volumetric flask, diluted with 5 mL methanol, shaken thoroughly, and then completed to the mark using the same solvent.

An accurately measured volume of Linezolid I.V solution equivalent to 10.0 mg was transferred into a 10 mL volumetric flask, 5 mL methanol was added, shaken well, and diluted to the required volume using the same solvent.

Accurately weighed ten Xarelto^®^ film-coated tablets were pulverized into fine powder then a weighed quantity equivalent to 10.0 mg of rivaroxaban was transferred into a 10 mL volumetric flask. 5 mL of acetonitrile was added, and the solution was sonicated for 10 min. Using the same solvent, the volume was completed to 10 mL, then the solution was filtered using a 0.45 µm membrane filter.

To prepare working solutions, serial dilutions of each stock solution (1.0 mg/mL) were diluted with methanol. The corresponding regression equations for each drug were used to estimate the % recoveries of REM, LNZ, and RIV.

#### Spiked human plasma

Human plasma samples of 1.0 mL were transferred to 15 mL centrifuge falcon tubes, spiked with various concentrations of the studied drugs. After completing the volume to 5 mL with acetonitrile as a protein precipitating agent, the solutions were vortexed for 1 min before centrifuging at 3600 rpm for 30 min. Concentration ranges of 20–550, 20–450, and 10–300 µg/mL for REM, LNZ, and RIV, respectively, was obtained by transferring 1.0 mL of clear supernatants into 10 mL volumetric flasks and making up to the mark with methanol. REM, LNZ, and RIV concentration ranges of 0.20–5.50, 0.20–4.50, and 0.1–3.00 µg/band, respectively, were obtained by applying 10 µL aliquots of each concentration flask onto the TLC plates in triplicate. A blank experiment was run, and calibration graphs were generated concurrently.

### Ethics approval

There are no human subjects in this research and informed consent is not applicable. The used plasma is pooled plasma from the blood bank of Mansoura university hospital.

## Results and discussion

Rapid and economic TLC- densitometric technique was optimized to develop a validated, sensitive, and highly selective method for the estimation of REM, LNZ and RIV with minimal environmental damage. The proposed TLC approach has the plus of simultaneously determining multi analytes with a very straightforward sample preparation process and low solvent consumption. Notably the proposed method offers several advantages over previously reported traditional HPLC techniques^17,29–33^, regarding its simplicity, cost-effectiveness, and rapid analysis time. Providing a promising alternative to conventional HPLC techniques for the estimation of REM in the presence of LNZ and RIV. Different chromatographic conditions were adjusted to achieve good separation with high resolution, sharp symmetric peaks and satisfactory R_f_ values.

### Method development and optimization

Different experimental parameters that affect the suggested TLC-densitometric technique such as mobile phase composition, saturation time, and scanning wavelengths, were adjusted.

First, a mixture of petroleum ether and ethyl acetate in different ratios was examined. LNZ and RIV separated well, but RIV and REM separated poorly.

Several solvent mixtures, such as ethyl acetate-methanol, ethyl acetate-ethanol, dichloromethane-methanol, dichloromethane-ethanol, dichloromethane-ethyl acetate and dichloromethane-acetone, were examined to enhance the separation.

Testing the ethyl acetate-methanol combination with different ratios, only LNZ and RIV were well resolved but RIV and REM were slightly resolved. Upon replacing methanol with ethanol, the mixture afforded merely resolved peaks with poor resolution.

Upon testing dichloromethane-methanol only REM was eluted, and the others were retained. While dichloromethane-ethyl acetate resulted in poor separation between LNZ and RIV. On the flip side, dichloromethane-ethanol has resulted in well resolved peaks however the REM peak has a tail. Thereby, acetic acid, formic acid, 25% ammonia solution, and triethylamine in different proportions were added to the tested mixture, however, poor resolution resulted. Eventually, a mixture of dichloromethane-acetone in different proportions was tested, yielding well-resolved sharp peaks with improved resolution. Therefore, mixture of dichloromethane-acetone in a ratio of 8.5:1.5 v/v was used throughout this approach.

Saturation times ranging from 15 to 45 min, were investigated since they had a considerable impact on chromatographic separation. With a saturation time of 30 min, satisfactory results were obtained. Different scanning wavelengths (235, 254, 280 nm) were tested, and 254 nm demonstrated the best sensitivity, yielding sharp, symmetrical peaks with minimal noise.

**Figure 2:**
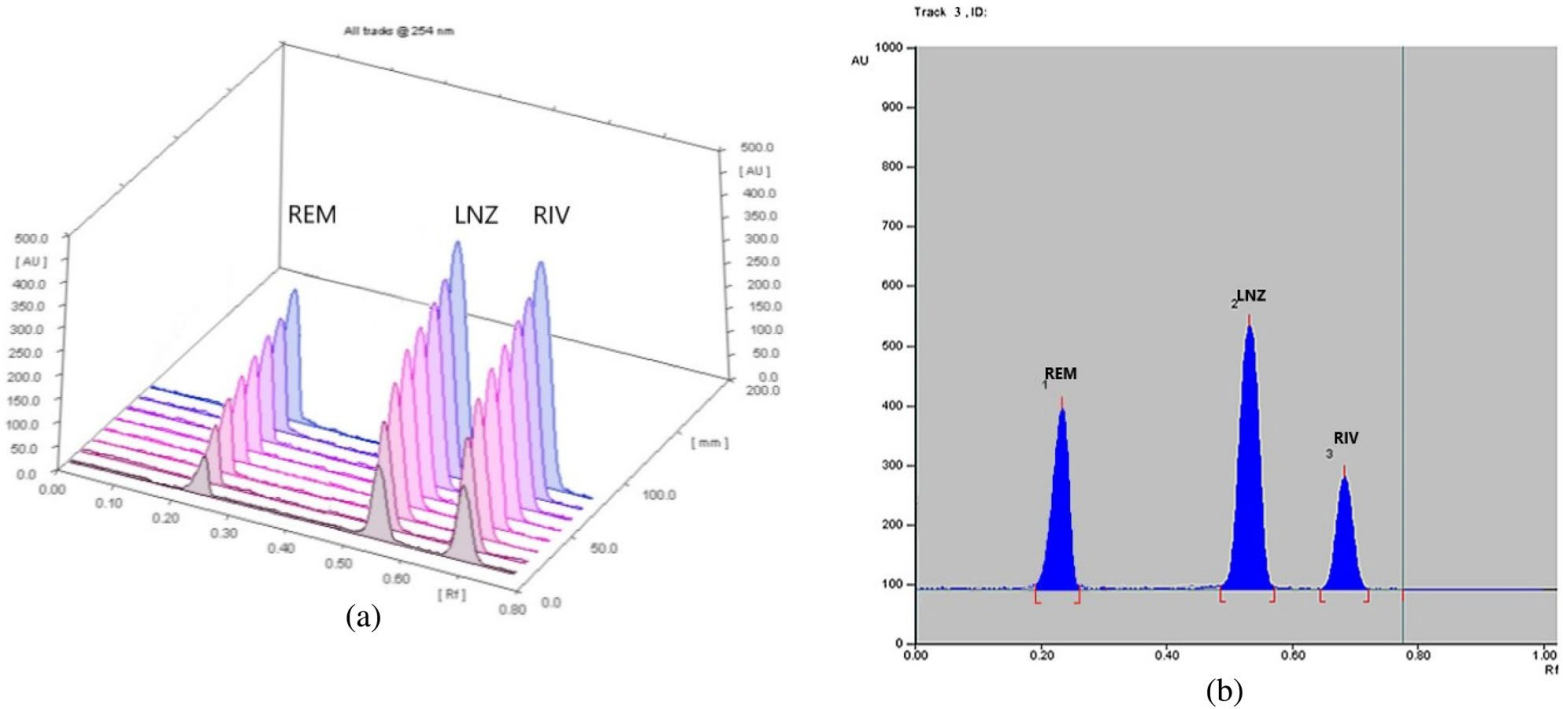
Densitogram of REM, LNZ and RIV separation by the proposed TLC method (**a**) 3D, (**b**) 2D.

**Figure 3:**
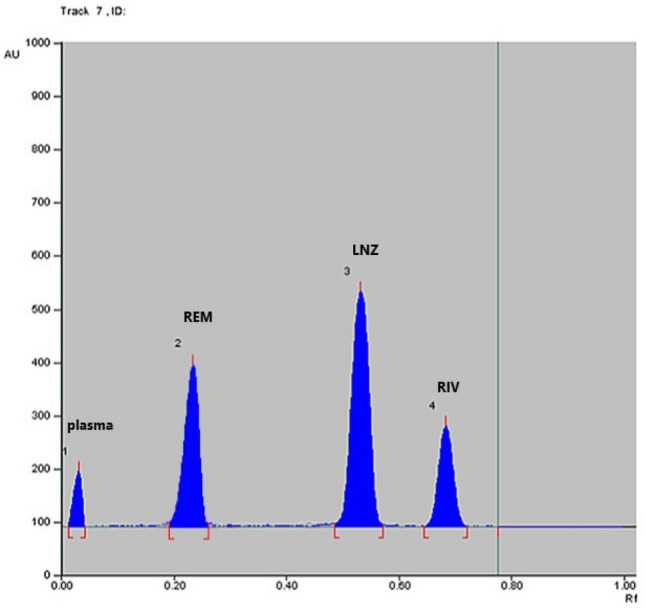
2D Densitogram of spiked human plasma samples.

Finally, dichloromethane–acetone (8.5:1.5, v/v) mixture was chosen as the eluent at scanning wavelength 254 nm. The examined drugs were separated with acceptable R_f_ of 0.04, 0.23, 0.53, 0.72 for plasma, REM, LNZ and RIV respectively, (Figs. 2 and 3).

### Method validation

ICH Q2(R1) recommendations have been implemented to evaluate the method’s validation criteria^34^, including:

### Linearity and range

Under the above mentioned densitometric procedures, the regression plots were generated over the concentration range of 0.20–5.50, 0.20–4.50 and 0.10–3.00 μg/band for REM, LNZ and RIV, respectively. Good correlation coefficients were obtained, and linear regression equations were as follow:

$$\left( {\text{For REM}} \right){\text{ y1}}\, = \,{2934}.{\text{4x}}\, + \,{1515}.{\text{9 R}}^{{2}} \, = \,0.{9998}.$$

$$\left( {\text{For LNZ}} \right){\text{ y2}}\, = \,{4}0{13}.{\text{5x}}\, + \,{1795}.0{\text{ R}}^{{2}} \, = \,0.{9999}.$$

$$\left( {\text{For RIV}} \right){\text{ y3}}\, = \,{52}00.{\text{2x}}\, + \,{2979}.{\text{5 R}}^{{2}} \, = \,0.{9998}.$$

where, y is the peak area at 254 nm, x is the concentration in µg/band, and R^2^ is the correlation coefficient. As illustrated by Table 1, the suggested technique has good linearity.

**Table 1:** Analytical performance data for the determination of the studied drugs by the proposed method.

Parameter	Pure analyte		
	REM	LNZ	RIV
Linearity range (µg/band)	0.20–5.50	0.20–4.50	0.1–3.00
Determination coefficient (R^2^)	0.9998	0.9999	0.9998
Intercept (a)	1515.9	1795.0	2979.5
Slope (b)	2934.4	4013.5	5200.2
S.D. of residuals (S_y/x_)	89.95	57.95	80.38
S.D. of intercept (S_a_)	37.78	20.26	29.06
S.D. of slope (S_b_)	0.02	16.07	31.03
S.D	1.19	0.94	0.83
LOD (µg/band)	1.20	0.016	0.018
LOQ (µg/band)	0.49	0.050	0.055

### Accuracy

To evaluate the accuracy of the suggested method, the percentage recoveries of six concentrations of analytes within the linearity range were analyzed in triplicate. The obtained data has shown that the proposed method has a high degree of reliability and accuracy with small values of standard deviation (Table 2).

**Table 2:** Accuracy evaluation for the estimation of REM, LNZ, RIV by the proposed method.

	Proposed method			Comparison methods ^17,38,39^		
Parameter	Conc taken (µg/band)	Conc found (µg/band)	% found	Conc taken (µg/mL)	Conc found (µg/mL)	% found
Remdesivir	0.2	0.201	100.50	20.0	20.220	101.10
	1.0	0.996	99.60	40.0	40.120	100.30
	1.5	1.501	100.06	60.0	59.820	99.70
	2.5	2.506	100.24			
	3.0	2.993	99.76			
	5.5	5.560	101.09			
Mean ± SD			100.20 ± 0.53			100.36 ± 0.70
% RSD			0.53			0.70
t-test			0.37			
F-value			1.69			
Linezolid	0.2	0.202	101.00	2.0	1.999	99.98
	1.0	1.006	100.60	6.0	6.036	100.60
	1.5	1.501	100.06	12.0	12.144	101.20
	2.5	2.498	99.92			
	3.5	3.507	100.20			
	4.5	4.494	99.86			
Mean ± SD			100.27 ± 0.44			100.59 ± 0.61
% RSD			0.44			0.60
t-test			0.91			
F-value			1.89			
Rivaroxaban	0.1	0.101	100.49	5.0	5.037	100.75
	0.5	0.495	99.04	10.0	9.950	99.50
	1.0	1.010	101.05	20.0	20.260	101.30
	2.0	1.995	99.77			
	2.5	2.504	100.16			
	3.0	2.997	99.92			
Mean ± SD			100.07 ± 0.68			100.51 ± 0.92
% RSD			0.68			0.91
t-test			0.83			
F-value			1.83			

Each result is an average of three separate determinations.

The tabulated t and F values are 2.36 and 5.78, respectively, at *p* = *0.05,* Ref.^37^.

### Intra- and inter-day precision

The proposed method’s intra- and inter-day precision was investigated. For intra-day precision, three different concentrations of each drug were measured in triplicate on the same day, and three successive days for inter-day precision. This was supported by the relative standard deviation (%RSD) values which were less than 2%, as shown in Table 3.

**Table 3:** Precision results for the determination of the studied drugs by the proposed TLC method.

Parameters		REM			LNZ			RIV		
		0.2 µg/band	2.5 µg/band	5.5 µg/band	0.2 µg/band	1.0 µg/band	3.5 µg/band	0.5 µg/band	1.0 µg/band	2.5 µg/band
Intraday	1	99.70	100.16	99.80	99.80	100.83	100.15	99.22	99.36	100.8
	2	100.15	99.60	99.99	100.40	100.78	99.75	100.01	100.19	100.25
	3	100.50	100.50	100.33	100.66	101.45	100.29	99.25	99.41	100.16
	Mean	100.12	100.08	100.04	100.28	101.02	100.06	99.49	99.65	100.40
	SD	0.40	0.45	0.27	0.44	0.37	0.28	0.45	0.47	0.35
	%RSD	0.40	0.45	0.27	0.44	0.37	0.28	0.45	0.47	0.35
Interday	1	100.12	100.08	100.04	100.40	100.78	100.15	99.49	99.65	100.4
	2	99.77	99.12	99.40	99.24	100.41	99.68	99.04	100.29	99.94
	3	100.70	99.34	100.40	99.50	99.71	100.54	100.26	99.17	99.45
	Mean	100.04	99.51	99.94	99.70	100.30	100.12	99.59	99.70	99.93
	SD	0.70	0.50	0.51	0.608	0.543	0.43	0.62	0.56	0.48
	%RSD	0.70	0.51	0.51	0.61	0.542	0.43	0.62	0.56	0.48

### Detection and quantitation limits

The method’s sensitivity was evaluated by calculating detection and quantitation limits using the standard deviation of the intercept (σ) and the average slope (S); where LOD; (3.3*σ)/S and LOQ; (10*σ)/S, respectively. The resulting limits for REM, LNZ, and RIV are shown in Table 1 confirming that the proposed method has good sensitivity.

### Robustness

Minor but intentional changes were made to the chromatographic technique parameters, and the percentage relative standard deviation (% RSD) was used to assess the robustness. Slight alteration was done in the proportions of the mobile phase system; dichloromethane, acetone (8.5:1.5 ± 0.10 mL), and saturation time (30 ± 5 min). The findings demonstrated the approaches’ reliability and robustness, demonstrating that the tested factors had no apparent impact (Table 4).

**Table 4:** Robustness testing of the proposed method.

Parameters	REM		LNZ		RIV	
	RSD of peak area	R_F_ ± SD	RSD of peak area	R_F_ ± SD	RSD of peak area	R_F_ ± SD
Mobile phase composition [DCM:acetone] (8.4 : 1.6, 8.5 : 1.5, 8.6 : 1.4 v/v)	0.583	0.23 ± 0.015	0.619	0.53 ± 0.025	1.037	0.72 ± 0.03
Saturation time (25, 30, 35 min.)	0.638	0.23 ± 0.02	0.963	0.53 ± 0.03	0.771	0.72 ± 0.02

### System suitability testing

System suitability tests showed good results compared to reference values for parameters like capacity factor, tailing factor, and resolution^35,36^. (Table 5).

**Table 5:** System suitability parameters of the proposed method.

Parameter	Proposed TLC method				Reference values^35,36^
	REM	LNZ		RIV	
Tailing factor, T	1.05	0.95		1.06	~ 1
Capacity factor, k´	3.34	0.87		0.39	0–10
Resolution, Rs		3.57	2		> 1.5

### Application to pharmaceutical formulations

The pharmaceutical formulations of REM, LNZ, and RIV were successfully estimated employing the suggested technique. The obtained percentage recoveries were satisfactory. Using the variance ratio F-test and the student’s t-test^37^, the results were compared to previously reported methods^17,38,39^. No significant differences were found, demonstrating the absence of excipient interference. The findings were acceptable, proving the proposed method’s high accuracy, Table 6.

**Table 6:** Estimation of the investigated drugs in their pharmaceutical dosage form with statistical comparison to previously reported methods.

	Proposed method			Comparison methods^17,38,39^		
Parameter	conc. taken (µg/band)	conc.found (µg/band)	% found	conc. taken (µg/mL)	conc.found (µg/mL)	% found
Remdesivir ampoule (100mg/100mL)	1.0	1.003	100.39	10.0	9.890	98.90
	2.5	2.485	99.42	30.0	29.800	99.30
	3.0	2.989	99.66	60.0	60.080	100.13
Mean			99.82			99.44
± SD			0.51			0.63
% RSD			0.51			0.63
t-test			0.81			
F-value			1.54			
Rivaroxaban (10 mg/tablet)	0.10	0.099	99.10	10.0	9.950	99.50
	1.0	1.003	100.03	15.0	15.180	101.20
	2.0	2.007	100.30	20.0	20.180	100.90
Mean			99.81			100.53
± SD			0.629			0.91
% RSD			0.631			0.90
t-test			1.13			
F-value			2.07			
Linezolid injection (200 mg/100 mL-vial)	0.20	0.201	100.28	2.0	1.998	99.90
	1.0	1.008	100.80	4.0	4.020	100.50
	3.5	3.483	99.50	6.0	6.080	101.33
Mean			100.19			100.57
± SD			0.65			0.72
% RSD			0.65			0.71
t-test			0.68			
F-value			1.20			

Each result is an average of three separate determinations.

The tabulated t and F values are 2.77 and 19, respectively, at *p* = *0.05*, Ref.^37^.

### Application to spiked human plasma

In human plasma, the new technique showed great sensitivity. The outcomes indicate that the method can accurately detect the investigated drug concentrations in human plasma without any interference, as shown in Fig. 3 and Table 7.

**Table 7:** Application of the proposed method to the determination of REM concurrently with LNZ and RIV in spiked human plasma.

Parameter	REM			LNZ			RIV		
	Con taken (µg/band)	Conc found (µg/band)	%recovery	Conc taken (µg/band)	Conc found* (µg/band)	%Recovery	Conc taken (µg/band)	Conc found* (µg/band)	%recovery
	1.0	0.983	98.32	0.2	0.193	96.50	0.100	0.098	98.90
	2.5	2.492	99.68	2.5	2.515	100.60	0.125	0.124	97.50
	5.5	5.302	96.40	4.5	4.414	98.08	0.150	0.152	101.40
Mean			98.13			98.39			99.30
SD			1.65			2.07			1.93
%RSD			1.68			2.10			1.96

### Green assessment of the proposed method

Green analysis relies on the use of green chemicals, limited usage of hazardous ones with no waste production and reduced energy consumption. Eco-Scale technique, the Green Analytical Procedure Index (GAPI) metric, and the Analytical GREEnness metric approach and Software were investigated to evaluate the suggested TLC-densitometric method’s greenness^40–42^. The green profiles for the proposed method using the mentioned metrics are shown in Table 8.

**Table 8:** Green Assessment of the proposed TLC method by Analytical Eco-scale, GAPI and AGREE.

(a) Analytical eco scale	
Reagent/instrument	Penalty point
Dichloromethane	2
Acetone	4
TLC scanner	0
TLC autosampler	0
Occupational hazard	0
Waste	3
Total penalty points	∑9
Analytical eco-scale total score	91

(b) Green analytical procedure Index (GAPI)	(c) Analytical Greenness metric approach (AGREE)
	

### Analytical eco-scale assessment of the proposed method

Analytical Eco-scale is an easy-to-implement technique for quality control laboratory work. The analytical Eco Scale score is computed using the following formula: analytical Eco Scale score = 100-∑ penalty points. It is determined by allocating penalty points to each of the parameters of the outlined technique, including the amount of reagents, occupational hazards waste, and energy^40^. The analytical approach is recognized as an outstanding green analysis if the score is higher than 75. The designed TLC-densitometric technique has an Eco-Scale score of 91, as shown in Table 8a, indicating that the outlined analytical procedure is considered green.

### GAPI assessment of the proposed method

A relatively recent method for gauging greenness is the green analytical process index (GAPI), created by Plotka-Wasylka^41^. It follows the entire process, from sample collection through waste processing. It presents 15 items to be examined using three degrees of color: green, yellow, or red, where red represents bad impact, yellow for intermediate, and green for safe and low environmental dangers. This allows for a thorough examination of each stage in the analytical procedure. The proposed technique fulfilled the majority of GAPI criteria with only 3 red regions. The first was caused by offline sample collection, the second by using a non-green solvent to precipitate plasma proteins, and the third by the absence of waste treatment possibilities. There are four yellow-colored regions corresponding to region 5, 10, 11 and 14. Region 5 is yellow as the method requires simple preparation such as filtration, while regions 10,11 are yellow because the solvents used have moderate health and environmental effects, according to the National Fire Protection Association (NFPA), and region 14 is yellow as the waste produced is less than 10 ml per sample. Overall, the results show that the suggested approach is environmentally friendly, as shown in Table 8b.

### AGREE assessment of the proposed method

Recently, the AGREE assessment tool was reported. The assessment is carried out with ease, implementing user-friendly software with an automatically created graph, and an evaluation report^42^. AGREE provides a clock-shaped graph with a perimeter divided into 12 sections based on the 12 tenets of green analytical chemistry. To assess whether the analytical technique complies with the green analytical chemistry (GAC) concept, each division is assigned to a different color on a red-yellow-green scale. A color and a number representing the overall evaluation on a scale of 0 to 1 are located in the center of the AGREE graph. The proposed method scored 0.81 as demonstrated in Table 8c, which indicates the greenness characteristics of the developed method.

## Conclusion

A simple, selective, and accurate chromatographic method has been developed for simultaneous determination of REM, LNZ and RIV in spiked human plasma. As multiple samples can be run simultaneously using minimal volumes of solvents, the TLC-densitometric approach saves time and lowers the cost of analysis. Moreover, the proposed method is eco-friendly with low impact on the environment. It has the benefits of being very selective, sensitive, and reproducible. It provides a fast and validated TLC analytical method for measuring remdesivir in the presence of linezolid and rivaroxaban for simple therapeutic medication monitoring and possible pharmacokinetic investigations.

## Data Availability

The datasets used and/or analysed during the current study available from the corresponding author on reasonable request.

## Abbreviations

LNZ: Linezolid
REM: Remdesivir
RIV: Rivaroxaban
TLC: Thin Layer Chromatography
R_f_: Retardation factors
